# A Longitudinal Correlational Study of Psychological Resilience, Depression Disorder, and Brain Functional–Structural Hybrid Connectome in Breast Cancer

**DOI:** 10.1155/2024/9294268

**Published:** 2024-11-18

**Authors:** Mu Zi Liang, Jin Zhou, Peng Chen, Ya Lan Song, Shu Han Li, Yu Yan Liang, Guang Yun Hu, Qu Hu, Zhe Sun, Yuan Liang Yu, Alex Molassiotis, M. Tish Knobf, Zeng Jie Ye

**Affiliations:** ^1^Department of Sexual and Reproductive Health, Guangdong Academy of Population Development, Guangzhou, China; ^2^Nursing Department, Southern Medical University Hospital of Integrated Traditional Chinese and Western Medicine, Guangzhou, China; ^3^Basic Medical School, Guizhou University of Traditional Chinese Medicine, Guiyang, China; ^4^Affiliated Cancer Hospital and Institute, Guangzhou Medical University, Guangzhou, China; ^5^School of Nursing, Guangzhou University of Chinese Medicine, Guangzhou, China; ^6^Shenzhen Bao'an Traditional Chinese Medicine Hospital, Guangzhou University of Chinese Medicine, Shenzhen, China; ^7^School of Nursing, Army Medical University, Chongqing Municipality, China; ^8^Department of Radiation Oncology, Chongqing University Cancer Hospital, Chongqing Municipality, China; ^9^The First Affiliated Hospital, Guangzhou University of Chinese Medicine, Guangzhou, China; ^10^Mental Health Education and Counseling Center, South China University of Technology, Guangzhou, China; ^11^College of Arts, Humanities and Education, University of Derby, Derby, UK; ^12^School of Nursing, Yale University, Orange, Connecticut, USA; ^13^School of Nursing, Guangzhou Medical University, Guangzhou, Guangdong, China

**Keywords:** Be Resilient to Breast Cancer, brain functional–structural hybrid connectome, breast cancer, depression disorder, psychological resilience

## Abstract

**Purposes:** To evaluate the association between psychological resilience, depression disorder (DD), and brain functional–structural hybrid connectome in patients with breast cancer before treatment (T0) and at 1 year.

**Methods:** Between February 2017 and October 2019, 172 patients were longitudinally enrolled from a multicenter trial named as Be Resilient to Breast Cancer (BRBC) and completed resting-state functional magnetic resonance imaging (rs-fMRI) and diffusion tensor imaging (DTI) before the T0. Data-driven multivoxel pattern analysis (MVPA) and correlational tractography (CT) were performed to identify distinct functional-structural hybrid connectome. DD was diagnosed by psychiatry physicians according to Diagnostic and Statistical Manual of Mental Disorders, Fifth Edition (DSM-5). Psychological resilience was collected by Resilience Scale Specific to Cancer (RS-SC) and tested as the mediation variable between hybrid connectome and DD.

**Results:** Of the total sample of 172, 14.5% (*N* = 25) were diagnosed with DD. High psychological resilience was associated with a lower risk of DD (hazard ratio (HR) = 0.37, 95% confidence interval (CI), 0.17–0.82, *p*=0.0368). Frontal pole right (88.0%) in rs-fMRI and arcuate fasciculus_L (75.2%) in DTI were identified as main significant brain areas. Psychological resilience accounted for 10.01%–12.14% of direct effect between brain functional–structural hybrid connectome and 1-year DD.

**Conclusion:** Psychological resilience predicts DD at 1 year and mediates the association between brain functional–structural hybrid connectome and DD at 1 year in patients diagnosed with breast cancer.

**Trial Registration:** ClinicalTrials.gov identifier: NCT03026374

## 1. Introduction

In 2022, breast cancer accounted for 11.6% of new cancer cases worldwide [1]. Due to early diagnosis and newer anticancer treatment (T0), the 5-year survival is approximating 90% in western countries and 80% in China, respectively [2]. Thus, attention has shifted to breast cancer survivorship and the quality of that survival [3]. There is evidence that depressive symptoms are prevalent in breast cancer and approximately 10%–25% of breast cancer survivors (BCSs) will be diagnosed with depression disorder (DD) especially in the first year following the diagnosis [4, 5]. Depression is associated with a poorer quality of life (QoL), lower adherence to medications, and a higher mortality risk [6–8]. Psychological resilience is defined as the capacity to thrive after traumatic events and is a robust buffer against negative emotions and behaviors across patients with different cancer types [9–11]. However, most research used a cross-sectional design and self-report instruments (i.e., patient health questionnaire-9 (PHQ-9)) instead of a clinical diagnosis by a provider. Psychological resilience and DD is not well explored in cancer with the use of imaging. In contrast, access to the neurobiological foundations for psychological resilience are well explored in different populations (i.e., young people/adults with childhood maltreatment) using noninvasive resting-state functional magnetic resonance imaging (rs-fMRI) and diffusion tensor imaging (DTI) [12, 13]. For example, middle frontal gyrus activation and greater functional connectivity related to cognitive control (i.e., inferior frontal gyrus, inferior parietal lobule) are recognized as strong predictors to nonrelapse (resilience) at 1-year follow-up in adults with a history of major depressive disorder (MDD) [14]. However, little is known about the longitudinal association between psychological resilience, brain functional–structural hybrid connectome, and DD in patients with breast cancer before and after T0. To address this gap, this study used data from a multicenter cohort named as Be Resilient to Breast Cancer (BRBC), a longitudinal study of newly diagnosed breast cancer patients before and at 1 year after diagnosis. Two data-driven methods, named as multivoxel pattern analysis (MVPA) for functional connectome and correlational tractography (CT) for structural connectome, were utilized to identify distinct patterns of functional-structural hybrid connectome against resilience group membership (high versus low resilience) [15, 16]. We hypothesized that: (1) psychological resilience would be a predictor of DD at 1 year, (2) distinct functional–structural hybrid connectome patterns would be recognized between high and low resilience groups, (3) psychological resilience would mediate the association between brain functional–structural hybrid connectome and DD at 1 year after diagnosis.

## 2. Method

### 2.1. Sample

**Figure 1:**
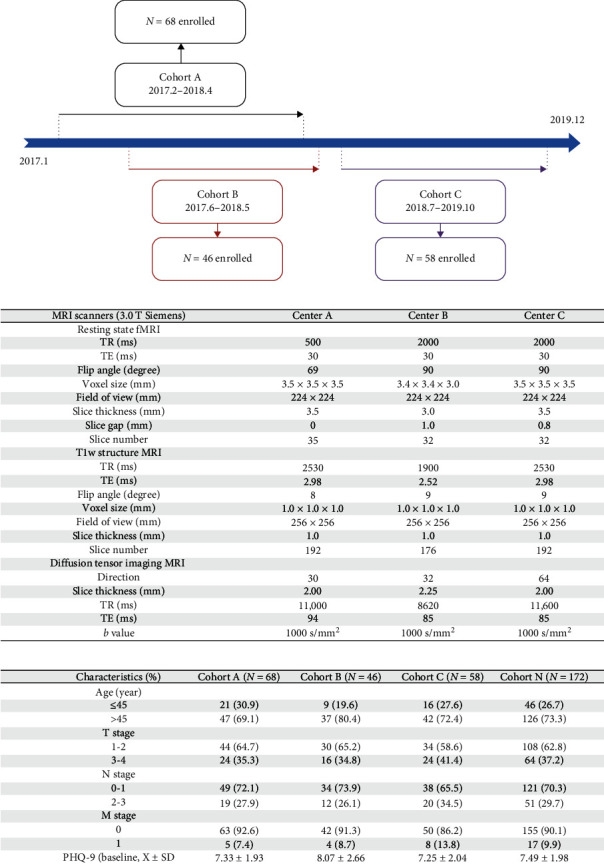
(A) Timeline for the enrollment in the BRBC. (B) rs-fMRI and DTI scanning parameters in different centers. (C) Baseline demographic and clinical characteristics for patients. BRBC, Be Resilient to Breast Cancer; DTI, diffusion tensor imaging; rs-fMRI, resting-state functional magnetic resonance imaging.

Between February 2017 and October 2019, 172 patients were enrolled in three cohorts over time in a multicenter trial named as BRBC [17–21]. The enrollment for the cohort was detailed in Figure 1A. The inclusion criteria for patients were: (1) aged >18 years, (2) fluent in Mandarin or Cantonese, (3) informed consent. The exclusion criteria were: (1) life expectancy less than 12 months, (2) declined to participate in the current study, (3) with active psychiatry disorders, which was detailed elsewhere [22–24]. Before the anticancer T0, patients completed the rs-fMRI and DTI. In addition, at T0, psychological resilience and self-reported depression were collected and DD was recorded by psychiatry physicians in the first year since the diagnosis. Informed consent was obtained from all patients and this multicenter trial was approved by the participating hospitals (2016KYTD08).

### 2.2. Data Collection

#### 2.2.1. 10-item Resilience Scale Specific to Cancer (RS-SC-10)

RS-SC-10 is derived from the original 25-item RS-SC-25 by using multidimensional item response theory (MIRT) [25–27]. Its psychometrics and invariance measurement have been well established in Chinese and American patients with different cancer diagnoses [28]. A 5-point Likert scaling is utilized with higher scores indicating higher resilience levels (score ranges from 10 to 50). The Cronbach's *α* was 0.88 in the present study. In addition, based on the standardized scores of RS-SC-10, a composite index named as resilience index (RI) was developed as a predictor of DD at 1 year in breast cancer by using the principal component analysis (PCA) [29]. The details about RI have been described elsewhere [23].

#### 2.2.2. PHQ-9

It has nine items about depressive symptoms based on the Diagnostic and Statistical Manual of Mental Disorders (DSM)-IV criteria [30]. The total score ranges from 0 to 27 with a higher score indicating a higher level of depressive symptom. In the current study, the Cronbach's *α* was 0.84.

#### 2.2.3. Brain Functional–Structural Hybrid Connectome Acquisition

3.0 T Siemens scanners were administered to collect rs-fMRI and DTI data across three centers (center A, B, and C) and the parameters for each scanner were detailed in Figure 1B. Spatial preprocessing procedures (i.e., smoothing and timing correction) were performed for rs-fMRI and 0.01 Hz < *f* < 0.10 Hz was utilized as the bandpass filter. As for DTI, movement and eddy current distortion were addressed before the fiber tracking algorithm.

### 2.3. Data Analysis

First, X-tile software was used to recognize the best cutoff of RI against the primary outcome of 1-year DD [31]. Based on the optimal cutoff of RI, the patients were divided into high versus low resilience groups and followed by a Kaplan–Meier analysis. Second, a data-driven MVPA was used to recognize significant functional connectome against the resilience group membership (high versus low) after controlling the confounders (i.e., center assignment, age, tumor node metastasis (TNM) staging, and PHQ-9) [32]. A conservative ratio of 40:1 was determined to retain the optimal component of MVPA taking the total cohort (*N* = 172) as the development dataset. Third, a data-driven CT using deterministic fiber tracking algorithm was utilized to identify quantitative anisotropy (QA) of significant structural connectome against the resilience group membership (high versus low) after controlling the confounders (i.e., center assignment, age, TNM staging, and PHQ-9) [33]. A 2.0 T threshold and a minimum length of 20 voxels with 4000 permutations were used to retain robust tracking fibers. A false discovery rate (FDR) < 0.05 was controlled for all rs-fMRI and DTI-related analysis. Fourth, a mediation analysis was performed between brain functional–structural hybrid connectome and 1-year DD after controlling the confounders (i.e., center assignment, age, TNM staging, and PHQ-9). In addition, the incremental prediction values of brain functional–structural hybrid connectome were estimated over a conventional prediction model using net reclassification improvement (NRI) and integrated discrimination improvement (IDI) according to the transparent reporting of a multivariable prediction model for individual prognosis or diagnosis (TRIPOD) guideline [34]. Conn toolbox (CONN) software, statistical parametric mapping (SPM) 12 and diffusion spectrum imaging (DSI) studio were utilized for brain functional–structural hybrid connectome and R software was utilized for mediation analysis and prediction models.

## 3. Results

### 3.1. Characteristics for the Cohort

The total sample was reflected in three cohorts: Cohort A (*N* = 68), Cohort B (*N* = 46), Cohort C (*N* = 58), who completed the rs-fMRI and DTI imaging at baseline (before treatment) and were followed up in the first year since the breast cancer diagnosis (Figure 1C). There was a small number lost to follow-up (7%) and the censoring time was recorded. No significant difference was identified between those lost to follow up and those who completed data collection (all *p*  > 0.05).

### 3.2. RI and DD

**Figure 2:**
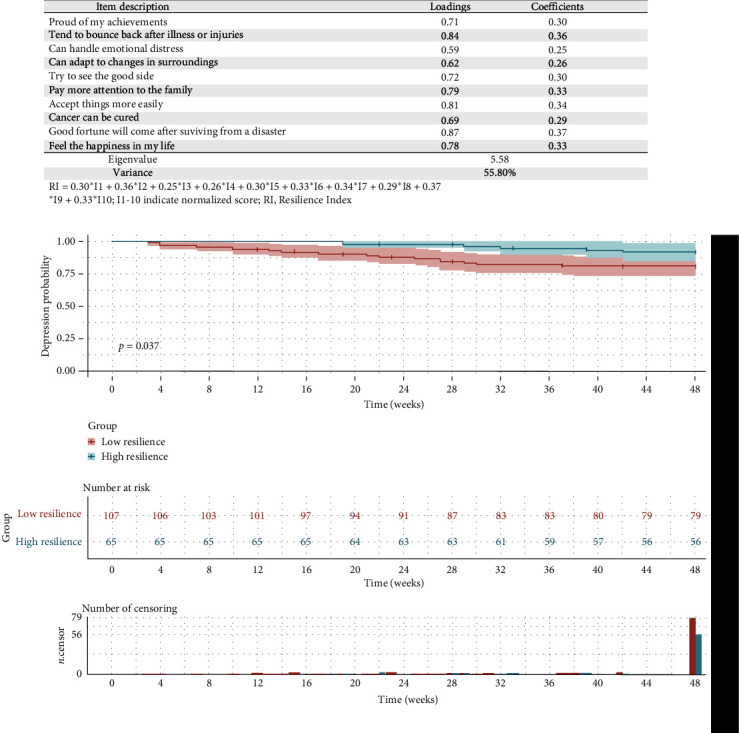
(A) PCA for RI. (B) The Kaplan–Meier analysis between psychological resilience group membership (high versus low) and 1-year DD. DD, depression disorder; PCA, principal component analysis; RI, resilience index.

Of the sample of 172, 14.5% were diagnosed with DD in the first year. In Figure 2A, the standardized calculation for RI was detailed and the optimal cutoff for RI was 1.72 by X-tile. Thus, 62.2% (*N* = 107) were classified as the low resilience group and 37.8% (*N* = 65) were classified as high resilience group. In Figure 2B, high psychological resilience was associated with a lower risk of DD (hazard ratio (HR) = 0.37, 95% confidence interval (CI), 0.17–0.82, *p*=0.0368).

### 3.3. MVPA and Significant Functional Connectome

**Figure 3:**
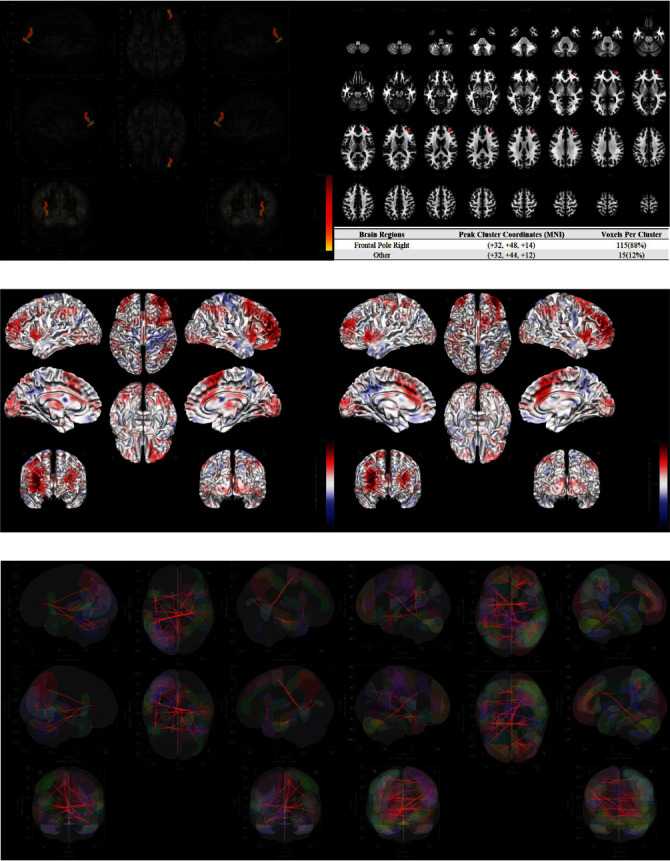
(A) Significant brain regions and voxels in MVPA. (B) Seed-to-voxel associations in the high/low resilience groups. (C) ROI to ROI associations in the high/low resilience groups. MVPA, multivoxel pattern analysis; ROI, region of interest.

In Figure 3A, based on the resilience group membership, frontal pole right (Montreal Neurological Institute [MNI] [+32, +48, +14], 115 voxels, 88%) was recognized as the significant brain functional areas by MVPA. The connectivities between MVPA-based region of interests (ROIs) and other brain areas in low and high resilience group were visualized in Figure 3B while the anatomical automatic labeling (AAL) based connectivities across the whole brain were visualized in Figure 3C.

### 3.4. CT and Significant Structural Connectome

**Figure 4:**
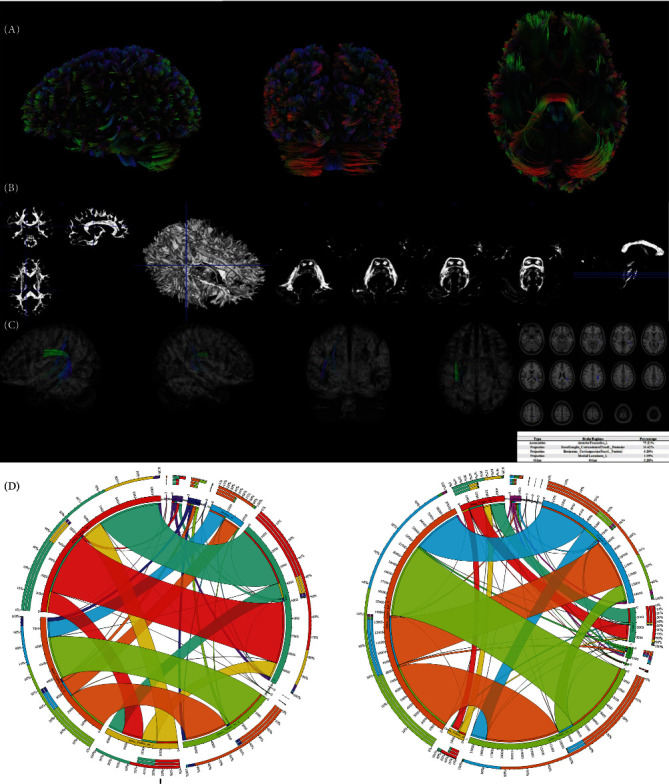
(A) Reconstruction for the fibers across the whole brain. (B) Super-resolution white matter imaging for all patients. (C) Significant neural pathways in CT. (D) ROI to ROI associations in the high/low resilience groups. CT, correlational tractography; ROI, region of interest.

In Figure 4A, a deterministic fiber tracking algorithm was used to reconstruct the white fibers. In Figure 4B, super-resolution white matter imaging for the total cohort was detailed. In Figure 4C, significant structural connectome was recognized between QA values and resilience group membership (high versus low) in Arcuate Fasciculus_L (75.2%), BasalGanglia_CorticostriatalTractL_Posterior (16.6%), Brainstem_CorticopontineTractL_Parietal (6.2%), and Medial Lemniscus_L (1.7%). The AAL-based connectivities across the whole brain in the two resilience groups were also presented in Figure 4D.

### 3.5. Mediation Analysis and Prediction Models

**Figure 5:**
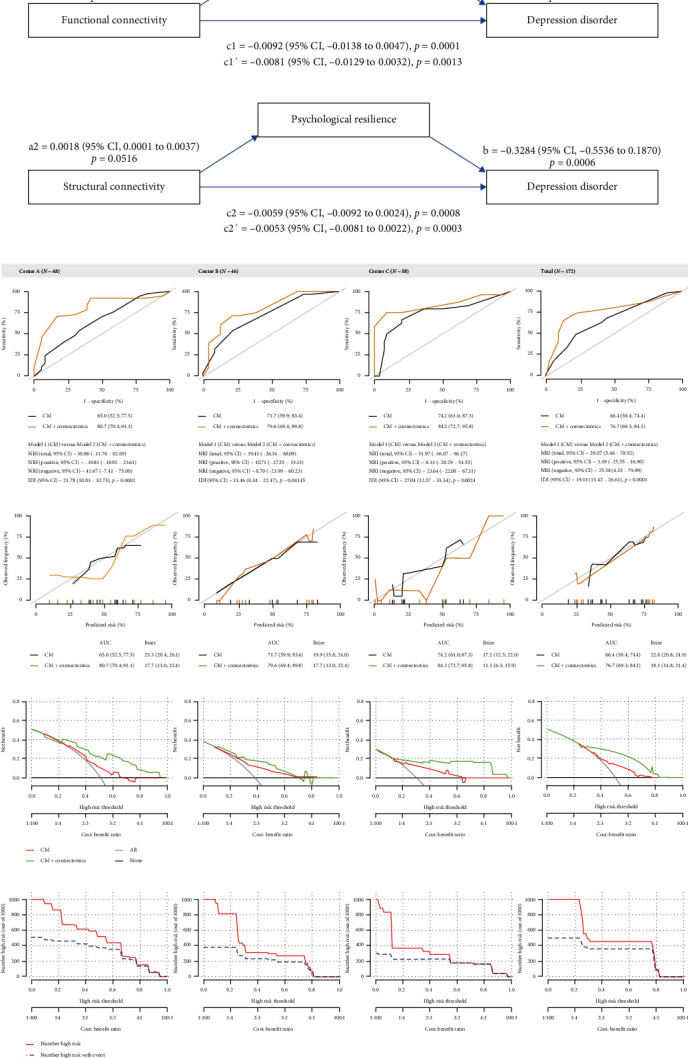
(A) The mediation analysis between brain functional–structural hybrid connectome and 1-year. (B) AUC, NRI, and IDI for Model 1 (CM) versus Model 2 (CM + connectomics). (C) Calibration curves for Model 1 (CM) versus Model 2 (CM + connectomics). (D) Decision curve analysis for Model 1 (CM) versus Model 2 (CM + connectomics). (E) Clinical impact curve for Model 2 (CM + connectomics). AUC, area under curve; CM, conventional model; DD, depression disorder; IDI, integrated discrimination improvement; NRI, net reclassification improvement.

In Figure 5A, the association between functional connectome and DD decreased from c1 = −0.0092 (95% CI, −0.0138 to −0.0047, *p*=0.0001) to c1′ = −0.0081 (95% CI, −0.0129 to −0.0032, *p*=0.0013) when psychological resilience was included. Similar findings were recognized in the association between structural connectome and DD (Figure 5A). The pathways of “Frontal Pole Right (88%)→resilience→depression disorder” and “Arcuate Fasciculus_L (75.2%)→resilience→depression disorder” were confirmed and psychological resilience accounted for 10.01%–12.14% of direct effect between brain functional–structural hybrid connectome and DD. In Figure 5B, area under curve (AUC) increased from 65.0% to 74.2% (Model 1: conventional model) to 76.7%–80.7% when functional–structural hybrid connectome were incorporated (Model 2: conventional model+ connectomics). NRI and IDI ranged from 19.41% to 31.97% and 11.46%–27.04%, respectively. In Figure 5C,a better fitting was recognized in Model 2 compared with Model 1 in consideration of Brier scores (11.1–18.1 Vs., 17.2–23.3). In Figure 5D, a higher net benefit was also identified in Model 2 compared with Model 1 and the clinical impact curve for Model 2 was presented in Figure 5E.

## 4. Discussion

This was the first study to perform a functional–structural hybrid connectome analysis using data-driven neuroimaging methods in breast cancer. In this longitudinal study, 14.5% patients with breast cancer were diagnosed with DD and psychological resilience was recognized as a strong predictor of DD at 1 year after initiation of treatment. Further, significant functional connectome in Frontal Pole Right, and structural connectome in Arcuate Fasciculus_L and BasalGanglia_CorticostriatalTractL_Posterior, were recognized between High and xLow resilience groups. At last, psychological resilience significantly mediated the association between brain functional–structural hybrid connectome and DD at 1 year after diagnosis.

First, previous cross-sectional research reported 10%–25% of BCS will be diagnosed with DD especially in the first year since the diagnosis, which was confirmed in the present study [4, 5]. According to the Kaplan–Meier analysis, psychological resilience was recognized as a strong predictor to 1-year DD and a similar association was also identified between psychological resilience and 1-year decreased QoL in breast cancer [23].

Second, without a priori ROI–ROI constraint, significant functional connectome in Frontal Pole Right was identified by a data-driven MVPA which was consistent with previous research that BCS were reported to have compromised cognitive function in brain frontal areas [35]. In addition, frontal medial cortex and frontal pole were also found to be associated with high-risk depression trajectories [36]. Further, significant structural connectome in Arcuate Fasciculus_L and BasalGanglia_CorticostriatalTractL_Posterior were identified by a data-driven CT. Interestingly, Arcuate Fasciculus (AF) links temporal regions to the inferior frontal gyrus and is often taken as one of the major language pathways in the human brain, while basal ganglia refers to a group of subcortical nuclei associated with reward processing and cognition control especially in motor control [37, 38]. In addition, Corticostriatal projections are essential components of forebrain circuits associated with motivated behavior [39]. These findings were not explored in previous research and might provide new insights about the association between psychological resilience and brain white matter. Of course, we should be noted that these structural pathways are reconstructed to connect vectors of water diffusion in each voxel which means the true axonal connections may not be “real” [40]. In combined consideration of small sample size in the current study, a large number of false positives should be noted although FDR was strictly controlled.

Third, psychological resilience accounted for 10.01%–12.14% of direct effect between brain functional–structural hybrid connectome and DD at 1 year and a similar mediation effect of psychological resilience was also recognized between default-mode network functional connectivity and COVID-19 vicarious traumatization [41]. Therefore, interventions to improve or strengthen psychological resilience could be a target mechanism for depressive symptoms. For example, supportive–expressive group therapy (SEGT) was associated with improved resilience and better survival [17, 18]. Based on the findings of rs-fMRI and DTI in the current study, a proof-of-concept randomized controlled trial with repeated MRI scanning should be considered to validate the causality between functional–structural hybrid connectome and psychological resilience and DD. Further, we found that incorporation of functional–structural hybrid connectome contributed incremental prediction values to DD and NRI and IDI ranged from 19.41% to 31.97% and 11.46%–27.04%, respectively. Thus, due to the noninvasiveness data collection by rs-fMRI and DTI, these neuroimaging methods could be utilized as a baseline vulnerability evaluation for DD but cost-effectiveness analysis should be further performed as these methods are relatively expensive [42].

## 5. Limitations

Several issues should be considered. First, patients were scanned by MRI only once before the treatment and we could not estimate the changes in brain functional–structural hybrid connectome which would be sensitive to chemo-, radio-, endocrine-related toxicity. A longitudinal design with repeated MRI and psychosocial screenings is recommended for future research. Second, without a priori constraint, data-driven methods could maximize the prediction ability of functional–structural hybrid connectome against DD at 1 year. However, sometimes significant brain areas identified by these methods could not be well explained. For example, 12% voxels could not be anatomically labelled in MVPA and similar issues should also be considered in CT. Third, in consideration of small sample sizes and unbalanced dataset (14.5% of DD incidence) cross different centers, the incremental prediction values of functional–structural hybrid connectome in the current study may not be well replicated in other populations with different cancer types. These findings should be further validated. At last, other confounders such as treatment modality as well as socioeconomic status (SES) are not controlled in the association estimation between resilience, DD, and brain connectome to avoid the overfitting issues. Thus, these potential confounders should be further tested in a larger sample.

## 6. Conclusion

Psychological resilience predicts DD at 1 year and mediates the association between brain functional–structural hybrid connectome and DD at 1 year in patients diagnosed with breast cancer.

## Data Availability

The data that support the findings of this study are available on request from the corresponding author. The data are not publicly available due to privacy or ethical restrictions.
